# Virtual multi-institutional tumor board: a strategy for personalized diagnoses and management of rare CNS tumors

**DOI:** 10.1007/s11060-024-04613-6

**Published:** 2024-03-01

**Authors:** James L. Rogers, Thomas Wall, Alvina A. Acquaye-Mallory, Lisa Boris, Yeonju Kim, Kenneth Aldape, Martha M. Quezado, John A. Butman, James G. Smirniotopoulos, Huma Chaudhry, Christina I. Tsien, Prashant Chittiboina, Kareem Zaghloul, Orwa Aboud, Nicholas G. Avgeropoulos, Eric C. Burton, David M. Cachia, Karan S. Dixit, Jan Drappatz, Erin M. Dunbar, Peter Forsyth, Edina Komlodi-Pasztor, Jacob Mandel, Byram H. Ozer, Eudocia Q. Lee, Surabhi Ranjan, Rimas V. Lukas, Margarita Raygada, Michael E. Salacz, Matthew A. Smith-Cohn, James Snyder, Ariane Soldatos, Brett J. Theeler, Brigitte C. Widemann, Kevin A. Camphausen, John D. Heiss, Terri S. Armstrong, Mark R. Gilbert, Marta Penas-Prado

**Affiliations:** 1 Neuro-Oncology Branch, Center for Cancer Research, National Cancer Institute, National Institutes of Health, 9030 Old Georgetown Rd, Bethesda, MD 20892 USA; 2 Laboratory of Pathology, Center for Cancer Research, National Cancer Institute, National Institutes of Health, 10 Center Dr, Bethesda, MD 20892 USA; 3 Radiology and Imaging Sciences, Clinical Center, National Institutes of Health, 10 Center Dr, Bethesda, MD 20892 USA; 4 Radiation Oncology Branch, Center for Cancer Research, National Cancer Institute, National Institutes of Health, 10 Center Dr, Bethesda, MD 20892 USA; 5 Proton Therapy Center, Sibley Memorial Hospital, Johns Hopkins Medicine, 5255 Loughboro Rd NW, Washington, DC 20016 USA; 6 Surgical Neurology Branch,, National Institute of Neurological Disorders and Stroke, National Institutes of Health, 10 Center Dr, Bethesda, MD 20892 USA; 7 Department of Neurology and Neurological Surgery, UC Davis Comprehensive Cancer Center, 4860 Y Street, Sacramento, CA 95817 USA; 8 Brain and Spine Tumor Program, Orlando Health Cancer Institute, 1400 S. Orange Ave, Orlando, FL 32806 USA; 9 Department of Hematology/Oncology, University of Massachusetts, 55 Lake Ave, Worcester, MA 01655 USA; 10 Lou and Jean Malnati Brain Tumor Institute, Northwestern University Feinberg School of Medicine, 675 N St Clair St, Chicago, IL 60611 USA; 11 Department of Neurology, University of Pittsburgh Medical Center, 5115 Centre Ave, Pittsburgh, PA 15232 USA; 12 Piedmont Brain Tumor Center, Piedmont Atlanta Hospital, Atlanta, GA 2001 Peachtree St30309 USA; 13 Department of Neuro-Oncology, Moffitt Cancer Center, 12902 USF Magnolia Drive, Tampa, FL 33612 USA; 14 Department of Neurology, MedStar Georgetown University Hospital, 3800 Reservoir Road Washington, Washington DC, 20007 USA; 15 Department of Neurology, Baylor College of Medicine, 7200 Cambridge St, Houston, TX 77030 USA; 16 Center for Neuro-Oncology, Dana Farber Cancer Institute, 450 Brookline Ave, Boston, MA 02215 USA; 17 Department of Neurology, Cleveland Clinic Florida, Weston Hospital, 2950 Cleveland Clinic Boulevard, Weston, FL 33331 US; 18 Eunice Kennedy Shriver National Institute of Child Health and Human Development, National Institutes of Health, 1 Center Dr, Bethesda, MD 20892 USA; 19 Department of Hematology and Medical Oncology, MD Anderson Cancer Center at Cooper, Cooper University Health Care, Two Cooper Plaza, Camden, NJ 08103 USA; 20 Benefis Sletten Cancer Institute, 1117 29Th St. S, Great Falls, MT 59405 USA; 21 Hermelin Brain Tumor Center, Henry Ford Cancer Institute, 2800 W Grand Blvd, Detroit, MI 48202 USA; 22 National Institute of Neurological Disorders and Stroke,, National Institutes of Health, 10 Center Dr, Bethesda, MD 20892 USA; 23 School of Medicine, Uniformed Services University, 4301 Jones Bridge Rd, Bethesda, MD 20814 USA; 24 Pediatric Oncology Branch, Center for Cancer Research, National Cancer Institute, National Institutes of Health, 10 Center Dr, Bethesda, MD 20892 USA; 25 Global Medical Affairs, Novocure GmbH, D4 Pk. 6, 6039 Root, Switzerland; 26 Wayne State University School of Medicine, 540 E Canfield St, Detroit, MI 48201 USA

**Keywords:** Collaborative practice, Multidisciplinary tumor boards, National Cancer Institute-Comprehensive Oncology Network Evaluating Rare CNS Tumors (NCI-CONNECT), Rare CNS tumors, Barriers to healthcare access

## Abstract

**Purpose:**

Multidisciplinary tumor boards (MTBs) integrate clinical, molecular, and radiological information and facilitate coordination of neuro-oncology care. During the COVID-19 pandemic, our MTB transitioned to a virtual and multi-institutional format. We hypothesized that this expansion would allow expert review of challenging neuro-oncology cases and contribute to the care of patients with limited access to specialized centers.

**Methods:**

We retrospectively reviewed records from virtual MTBs held between 04/2020–03/2021. Data collected included measures of potential clinical impact, including referrals to observational or therapeutic studies, referrals for specialized neuropathology analysis, and whether molecular findings led to a change in diagnosis and/or guided management suggestions.

**Results:**

During 25 meetings, 32 presenters discussed 44 cases. Approximately half (*n* = 20; 48%) involved a rare central nervous system (CNS) tumor. In 21% (*n* = 9) the diagnosis was changed or refined based on molecular profiling obtained at the NIH and in 36% (*n* = 15) molecular findings guided management. Clinical trial suggestions were offered to 31% (*n* = 13), enrollment in the observational NCI Natural History Study to 21% (*n* = 9), neuropathology review and molecular testing at the NIH to 17% (*n* = 7), and all received management suggestions.

**Conclusion:**

Virtual multi-institutional MTBs enable remote expert review of CNS tumors. We propose them as a strategy to facilitate expert opinions from specialized centers, especially for rare CNS tumors, helping mitigate geographic barriers to patient care and serving as a pre-screening tool for studies. Advanced molecular testing is key to obtaining a precise diagnosis, discovering potentially actionable targets, and guiding management.

**Supplementary Information:**

The online version contains supplementary material available at 10.1007/s11060-024-04613-6.

## Introduction

Multidisciplinary tumor boards (MTB) help coordinate [1–3] and deliver complex care to patients with central nervous system (CNS) tumors [4] and have been suggested as a quality metric by the American Academy of Neurology and Society for Neuro-Oncology [5]. Optimal care involves collaboration across specialties, including neuro-oncology, medical oncology, neuroradiology, neurosurgery, radiation oncology, neuropathology, and others. This diverse clinical expertise is often clustered in large academic centers, making MTBs mostly feasible within this environment. Virtual meetings provide a unique opportunity for participation of providers located at different institutions, facilitating the identification of unusual diagnoses and personalized management for patients who otherwise would have difficult in-person access to the expert care located in these tertiary or quaternary institutions.

Previous studies have explored the use of MTBs in breast [6], colorectal [7], esophageal [8], gynecological [9], head and neck [1], lung[10], upper-gastrointestinal [11], and urological [12] cancers, as well as in other mixed cancer cohorts [13, 14]. Their role in neuro-oncology has been explored in recent publications. In a US nationwide provider survey, Snyder et al. (2017) identified four benefits of neuro-oncology MTBs (coordination of care, direction for complicated cases, education, and communication of potential clinical trials) [4]. Khalafallah et al. (2020) quantified the utility of neuro-oncology MTBs reporting numerous diagnostic and treatment plan changes and shortened referral times [15]. Schäfer et al. (2021) administered a German nationwide provider survey, identifying that virtual neuro-oncology MTBs are feasible and acceptable [16]. Gaudino et al. (2022) described their experience holding neuro-oncology MTB meetings in Italy, noting the significance and limitations of incorporating imaging review by an expert neuroradiologist [17]. Lastly, a study by Ivanovic et al. at a tertiary academic center demonstrated a strong correlation between high MTB participation and low diagnostic error rates by neuroradiologists [18]. As suggested by these publications, MTBs are clinically impactful in neuro-oncology, benefiting both providers and patients.

However, while MTBs are common in specialized neuro-oncology centers, no study has yet analyzed their utility in 1) allowing virtual participation of multiple institutions, and 2) providing remote expert guidance for providers taking care of patients with rare CNS tumors, whose diagnosis and management are particularly challenging [19]. Few centers have the expertise to perform advanced molecular testing and/or develop tailored treatment plans for these uncommon tumors. At the National Cancer Institute (NCI), several authors of this manuscript participate in caring for patients with rare CNS tumors under the NCI’s Comprehensive Oncology Network Evaluating Rare CNS Tumors (NCI-CONNECT; https://ccr.cancer.gov/neuro-oncology-branch/connect), a program to advance the understanding of a pilot group of rare CNS cancers in adults, including atypical teratoid rhabdoid tumor (ATRT), choroid plexus tumors, histone mutated gliomas, ependymoma, gliomatosis cerebri, gliosarcoma and primary CNS sarcomas, medulloblastoma, high-grade meningioma, IDH mutated gliomas, parenchymal pineal region tumors, BRAF-altered tumors, embryonal tumors formerly known as primitive neuro-ectodermal tumors, and DNA methylation-defined emerging CNS tumors. In the US, these rare tumors have an estimated prevalence in the low hundreds up to < 14,000 individuals across all ages, and an incidence in adults ranging from a few tens to a little over a thousand new cases per year[20–25]. NCI-CONNECT facilitates referrals to a specialized quaternary center focused on clinical epigenetics and management of rare CNS tumors [26]. Specialized care can reduce the likelihood of misdiagnosis and treatment-related complications, resulting in more appropriate management and, ultimately, improved outcomes [27].

Review and discussion of cases among providers from different institutions can be accomplished remotely, facilitating referrals to tertiary and quaternary centers and circumventing typical geographic constraints and other barriers that limit obtaining second opinions from specialized centers [28]. In this study, we describe our experience of remotely reviewing neuro-oncology cases, with a primary focus on rare CNS tumors, during virtual MTBs hosted by the NCI Neuro-Oncology Branch and open to the participation of multiple institutions. While findings are reported in the context of the COVID-19 pandemic, we propose virtual multi-institutional tumor boards led by specialized centers as a strategy to optimize neuro-oncology clinical practice, especially for uncommon CNS tumors.

## Materials and Methods

### Overview of the Virtual MTB

At the National Institutes of Health (NIH), Neuro-Oncology MTBs were traditionally held in-person and focused on internal cases. In March 2020, due to the COVID-19 pandemic, the MTB was transitioned to a virtual format. This permitted expanding participation to multiple clinical teams from neighbor institutions and over 30 Brain Tumor Trials Collaborative (BTTC)/NCI-CONNECT network centers across the US, enabling the presentation of external cases. This virtual multi-institutional format continues at present.

### Preparation for the Virtual MTB

Before each MTB, presenters complete a standardized portable document format (PDF) data capture form (see Supplementary Material 1), inquiring about date of initial diagnosis, reason for discussion at MTB, and de-identified brief patient narrative (e.g., age, gender, handedness, current Karnofsky Performance Status (KPS) score, relevant symptoms and exam findings, and treatment summary). This form is emailed to MTB attendees to allow them to review the basic case details before MTB.

### During the Virtual MTB

Virtual CME-accredited neuro-oncology MTBs have been held since April 2020, 2–4 times per month, via the WebEx video teleconferencing platform (WebEx by Cisco, San Jose, California, US), vetted by the NIH Center for Information Technology to be secure for patient care discussions. Meetings are attended by a diverse group of clinicians, researchers, and trainees from the NIH and BTTC/NCI-CONNECT network and other external sites.

The presenter is asked to share a deidentified patient summary and display relevant imaging and pathology results including molecular data if already available. NIH neuroradiologists and neuropathologists comment on the data. NIH neuro-oncologists, neurosurgeons, radiation oncologists, a patient-reported outcomes expert, a genetic counselor, and other external clinicians, are invited to provide input on diagnosis and management strategies, including potential clinical trial options when applicable. MTBs are moderated to ensure adequate time and discussion for each case and summarize the suggestions for the presenter(s). Attendees are asked to keep all information confidential, and presenters are advised that the tumor board suggestions are meant to contribute, but not dictate, the final management of their cases.

### After the Virtual MTB

A PDF document is emailed to the presenter with a summary of the discussion and suggestions, including available research protocols. Presenters are encouraged to bring their cases back for follow-up when appropriate. Frequently, presenters are offered to refer their patients for an in-person visit under the NCI Natural History Study (NCT02851706; PI: T. S. Armstrong) and/or submit a request for neuropathology consultation at the NIH, particularly if the diagnosis was unclear based on histopathological features and available molecular data. For such reviews, presenters are asked to ship tumor tissue to the NCI Laboratory of Pathology for testing, including use of a custom next-generation DNA and RNA sequencing gene panel and DNA methylation-based classifier [29]. For patients referred for an in person visit, the observational NCI Natural History Study enables tumor tissue testing and longitudinal clinical tracking of patients throughout their disease, seeking to understand epidemiologic risk factors and tumor molecular features, and using clinical outcomes assessments to monitor the impact of the disease and treatment on how patients feel and function. Patients with rare CNS tumors also receive evaluation by a genetic counselor and have access to the Coping, Advocacy, Relationships, Education, and Support (CARES) group program, which supports wellness and creates connections among rare CNS tumor patients.

### Retrospective MTB Record Review

As of July 2023, a total of 154 cases have been presented (112 external, including 2 international, with presentations by 47 different external institutions). We retrospectively reviewed standardized PDF, medical, and email records from the first year of virtual MTBs (April 2020-March 2021). The following variables were extracted by J.L.R. and T.W., under the guidance of the senior author (M.P.P.): date of the presentation, presenter’s name and affiliation, patient’s age, pre-MTB diagnosis, timing along the disease trajectory (e.g., newly diagnosed, recurrent, other), tumor location, and presence of dissemination in the CNS or systemically (yes or no; locations).

Additionally, the suggestions offered during the presentation were collected, including: 1) in person participation in the NCI Natural History Study, 2) participation in a therapeutic clinical trial at the NIH and/or other institutions, 3) further neuropathology review and molecular testing at the NIH. Data collection also included: 1) if molecular profiling was performed by NIH (before or after MTB), 2) if molecular findings led to a change in diagnosis (new diagnosis or further sub-classification), and 3) if the molecular findings (obtained at the NIH or elsewhere) guided management suggestions (germline testing/genetic counseling, observation vs. treatment, or specific treatment options).

### Statistical Analyses and Ethical Approval

All data were analyzed using Microsoft Excel, version 16.59 (Microsoft Corporation, Redmond, Washington, US). Due to the number of cases and the retrospective nature of the review, only descriptive statistics were obtained and reported.

The National Institutes of Health (NIH) Office of IRB Operations (IRBO) determined that the study did not require IRB review or approval. Patient consent was waived because only deidentified data were discussed and extracted.

## Results

During 25 virtual meetings held between April 2020-March 2021, 32 providers from 18 institutions presented 44 cases (range: 1–4 cases/meeting). Of these cases, 12 (27%) were presented by an NIH clinician, 25 (57%) by an outside clinician, and 7 (16%) were mutual cases presented jointly by NIH and outside clinicians. Two out of these 44 cases (5%) were presented for their educational value and were not seeking discussion regarding diagnosis or management. Therefore, only 42 cases (with 1 case presented twice; 41 unique patients) were included for further analyses of MTB impact. Table 1 summarizes the patient’s age and the pre and post MTB diagnoses. Of these 41 unique patients, 13 had dissemination outside of the primary location (*n* = 6) and/or outside the CNS (*n* = 13). The median age was 40 years of age (range 15 to 73), with 20 (49%) being adolescents and young adults (AYA). At the time of MTB presentation, 36% cases (*n* = 15) were newly diagnosed, 38% (*n* = 16) had recurrence, and 26% (*n* = 11) were classified as “other,” indicating mainly lack of clarity between recurrence and treatment-related effects. Tumor types were 9 infiltrating gliomas, 8 ependymomas, 5 embryonal tumors, 4 circumscribed astrocytic gliomas, 3 glioneuronal tumors, 3 meningiomas, 3 mesenchymal, non-meningothelial tumors, 1 paraspinal nerve tumor, 1 sellar tumor, and 4 were non-classifiable or undiagnosed histology. Approximately half of the cases (*n* = 20, 48%) involved an NCI-CONNECT rare CNS tumor. At the time of presentation, 29% (n = 12) of patients were already enrolled on the NCI Natural History Study and 40% (*n* = 17) had already been reviewed by the NIH Laboratory of Pathology team (either as Natural History Study participants or as external consultations).

**Table 1:** Pre and post tumor board diagnoses after incorporation of molecular data obtained at the NIH

Patient age	Pre-tumor board diagnosis^*^	Post-tumor board change
43	Astrocytoma, WHO Grade 2	Yes—Subclassification (*IDH* mutant) Change in grade from 2 to 3
22	Astrocytoma, WHO Grade 3^#^	No
43	Glioblastoma, *IDH* mutant	No
NC	Glioblastoma, *IDH* wild-type, *MGMT* methylated	No
73	Glioblastoma, *IDH* wild-type	No
46	High-grade astrocytoma with molecular features of Glioblastoma	No
52	Infiltrating glioma, *IDH* wild-type	No
66	Diffuse midline glioma^&^, *H3K27M* mutant	No
36	Pilocytic astrocytoma with *BRAF* fusion	No
66	Infiltrating glioma, *IDH* wild-type, *BRAF* fusion	Yes—Revised diagnosis (Pilocytic astrocytoma)
27	Pleomorphic xanthoastrocytoma^&^, WHO Grade 3	No
29	Ganglioglioma	No
47	Diffuse leptomeningeal glioneuronal tumor	No
51	Rosette-forming glioneuronal tumor, WHO Grade 1	No
39	Ependymoma^&^, WHO Grade 3	No
27	Supratentorial *RELA*-fusion ependymoma^&^	No
67	Ependymoma^&^	Yes—Subclassification (PF-B)
50	Ependymoma^&^, WHO Grade 3	Yes—Subclassification (PF-B)
25	Ependymoma^&^, WHO Grade 3	Yes—Subclassification (MYCN amplified ependymoma)
30	*MYCN*-amplified anaplastic ependymoma^&^	No
47	Myxopapillary ependymoma^&^	No
42	Myxopapillary ependymoma^&^	No
47	Medulloblastoma^&^, SHH subgroup and pituitary mass (macroadenoma vs. metastatic medulloblastoma)	No
48	Medulloblastoma^&^	Yes—Subclassification (SHH subgroup)
40	Medulloblastoma^&^, SHH subgroup	No
37	High-grade neuroendocrine tumor/Primitive neuroepithelial tumor^&^	Yes—Revised diagnosis (Atypical teratoid/rhabdoid tumor, MYC subtype)
25	Atypical teratoid/rhabdoid tumor^&^	No
61	Malignant melanocytic schwannian tumor	No
40	Meningioma^&^ (WHO grade 1 vs. grade 2)	Yes—Subclassification (DNA Methylation show higher risk)
65	Meningioma^&^, WHO grade 1	No
57	Meningioma^&^(WHO grade 1 vs. grade 2)	No
30	Hemangioblastoma, WHO grade 1	No
NC	High-grade pleomorphic spindle cell sarcoma^3^	No
38	Myxoid mesenchymal tumor with *EWSR1-ATF1* fusion	No
21	Craniopharyngioma, adamantinomatous	No
18	Infiltrating glioma; *IDH1*, *BRAFV600E* negative; *H3K27me3* retained	No
40	High-grade glioma with ependymal and sarcomatous differentiation	No
35	CNS embryonal tumor with rhabdoid features^&^	Yes—Revised diagnosis (High-grade neuro-epithelial tumor with *MN1* alteration)
54	Undiagnosed (brain lesions in patient with Hodgkin’s lymphoma)	No
15	Undiagnosed (fibrous lesion vs. low grade sarcoma)	No
17	Undiagnosed (inflammatory non-neoplastic entity)	No

^*^Tumors were presented and therefore classified before the release of the latest 2021 WHO Classification of Tumors of the Central Nervous System [35]. The current designation of Glioblastoma, *IDH*-mutant is Astrocytoma, *IDH*-mutant, WHO grade 4. Supratentorial *RELA-*fusion ependymoma is now Supratentorial ependymoma, *ZFTA* fusion-positive

^#^This case was presented to the tumor board twice

^&^NCI-CONNECT tumor type

*Abbreviations*: IDH, Isocitrate Dehydrogenase; LMD, leptomeningeal disease; MCYN, V-Myc Avian Myelocytomatosis Viral Oncogene Neuroblastoma Derived Homolog; MGMT, O^6^-Methylguanine-DNA Methyltransferase; N/A, Not Applicable; NCI-CONNECT, National Cancer Institute-Comprehensive Oncology Network Evaluating Rare CNS Tumors; NC, Not Collected; RELA, V-Rel Avian Reticuloendotheliosis Viral Oncogene Homolog A; SHH, Sonic Hedgehog; WHO, World Health Organization

As displayed in Table 2, 31% of cases (*n* = 13) received a recommendation for participation in therapeutic clinical trials. Of these patients, 8 were potentially eligible for trials at the time of MTB presentation, including trials enrolling at the NIH (*n* = 5), an external institution (*n* = 1), or both NIH and an external institution (*n* = 2). Five additional patients were potentially eligible for NIH trials at the time of future disease progression.

**Table 2:** Summary of tumor board suggestions

Tumor board suggestion or outcome	N	%
Offered participation in observational NCI Natural History Study*	9	21.4
Offered neuropathology review and molecular testing at NIH	7	16.7
Offered participation in therapeutic trial now or if future progression/recurrence:	13	31.0
Trial at NIH, now**	7	16.7
Trial at external institution, now**	3	7.1
Trial at NIH, if future progression/recurrence	5	11.9
Cases where molecular findings# guided management:	15	35.7
Germline testing/counseling recommended	3	7.1
Observation vs. treatment recommended	3	7.1
Specific treatment options recommended	11	26.2

^*^ NCT02851706

^**^Two patients received suggestions for trials both at NIH and an external site. Eight patients received trial suggestions for current clinical needs

^#^Molecular findings available at the time of tumor board presentation, obtained at the NIH or outside. More than one recommendation was given in one case (i.e., both germline testing/counseling and observation vs. treatment)

*Abbreviations*: NCI, National Cancer Institute; NIH, National Institutes of Health

Additionally, of the total 42 cases discussed, 21% (*n* = 9) were offered enrollment in the NCI Natural History Study, 17% (*n* = 7) were recommended neuropathology review at the NIH, including advanced molecular testing due to diagnostic uncertainty, and 100% (*n* = 42) received suggestions for further management.

In 52% of cases (*n* = 22) molecular profiling occurred at the NIH, either before or after MTB presentation. In 15 cases, molecular findings available during presentation (either obtained at the NIH or outside), guided MTB management suggestions in one or more of these areas: 1) observation versus treatment (*n* = 3); 2) specific treatment selection (*n* = 11); 3) recommendation to proceed with germline testing and/or genetic counseling (*n* = 3) (Table 2). Lastly, MTB discussions resulted in 9 (21.4%) cases having a diagnosis change (Table 1), including identification of an entirely new diagnosis (*n* = 3: from infiltrating glioma to pilocytic astrocytoma; from primitive neuroepithelial tumor to AT/RT; and from CNS embryonal tumor with rhabdoid features to astroblastoma, *MN1*-altered) or further subclassification (*n* = 6: identified molecular subgroup in 3 ependymomas and 1 medulloblastoma; 1 infiltrating astrocytoma classified as *IDH*-mutant and increased grade; and 1 meningioma classified as higher risk based on DNA methylation). Supplementary Table 1 provides a summary of all the cases for which molecular data obtained at the NIH and/ or elsewhere was important in guiding diagnosis or the suggestions of the MTB. This supplementary table includes the listing of the molecular aberrations, methods used, if diagnosis was changed or further classified, a summary of the suggestions provided by the MTB and evidence level.

## Discussion

MTBs can optimize the delivery of complex cancer care [13]. We report our experience of organizing a regular virtual MTB with participants from multiple institutions for discussion of complex neuro-oncology cases including rare CNS tumors. The results of a retrospective record review suggest that the virtual MTB is an effective forum for discussions of molecular, radiological, and clinical information for personalized diagnoses and patient management suggestions, particularly for patients with rare CNS tumors for which specialized care can be difficult to access.

Opening the meetings to outside participation fostered collaboration among providers from multiple institutions throughout the US, as illustrated by the mutual cases presented jointly by NIH and outside clinicians. Additionally, similar to the tumor board conferences outlined by Snyder et al. (2017) [4], our virtual MTBs involved members of diverse subspecialties. Engaging a multidisciplinary audience from multiple institutions enabled different specialists to inform management, a crucial benefit of team-based virtual tumor board meetings [30].

Unfortunately, there are many barriers to specialized care and enrollment in therapeutic clinical trials in neuro-oncology. As Lee et al. (2019) outlined, physician-related logistical barriers may impede referral to clinical trials, among multiple other factors [31]. In our virtual MTB, almost 1/3 of presented cases received therapeutic clinical trial suggestions. Additionally, 21% (*n* = 9) were offered enrollment in the observational NCI Natural History Study, providing an opportunity to collect prospective data in rare CNS tumors. As Rogers et al. (2020) noted in their survey of the Society for Neuro-Oncology members, the most commonly reported provider-referral barrier was finding trials in the patient’s geographic area [32]. In our experience, virtual MTB meetings with colleagues from across the US aided in identifying appropriate therapeutic or observational studies in which patients could realistically enroll.

In recent years, objective and biologically-driven methods for classification of CNS tumors have been increasingly adopted [33]. The cIMPACT-NOW group (Consortium to Inform Molecular and Practical Approaches to CNS Tumor Taxonomy) pushed for an updated classification considering the rapid progress in molecular insights into these neoplasms [34]. Notably, the new fifth edition of the WHO Classification, published in 2021, advances the role of molecular testing and emphasizes the importance of integrated diagnoses [35]. In the new “molecular era” of neuro-oncology, targeted next-generation sequencing [36] and DNA methylation profiling, particularly the DKFZ/Heidelberg CNS tumor classifier [29, 37], have emerged as important precision diagnostic tools [33] that directly impact patient care [38]. In diagnostically complex neuro-oncology cases (especially those involving rare CNS tumors), methylation profiling has been particularly diagnostically useful [39].

Neuropathologic review by the NIH and available molecular findings discussed during MTBs guided management, including observation versus treatment, specific treatment selection, and recommendation for germline testing and/or genetic counseling. Therefore, molecular findings were leveraged to achieve accurate, personalized diagnoses and develop individualized patient management. Based on our experience, molecular profiling is particularly valuable for any cases with equivocal or insufficient histopathological features for proper classification into a defined WHO category or subcategory. For example, astroblastomas-MN1 altered, molecular subtypes of ependymoma and medulloblastoma subgroups require this advanced testing.

All cases presented at MTB received suggestions for management, with most providers reporting, via quality improvement surveys (not reported here), that the suggestions impacted their patient management. Virtual MTB discussions also resulted in diagnosis changes for 21% of cases, including identification of entirely new diagnoses and further subclassification. This finding highlights the importance of referring patients to highly specialized neuro-oncology centers, which leverage advanced molecular testing as a complement to traditional histology, have rich experience in imaging interpretation [40], and incorporate a robust basic, translational, and clinical research program [41].

The virtual MTB was particularly impactful for those with ependymomas and other rare CNS tumors that are infrequently seen by most clinicians, even in academic centers. Approximately half of the presented cases involved the diagnosis and management of an NCI-CONNECT rare CNS tumor and the median age was young. While many of the rare tumors presented at the MTB still lack well-defined standards of care [26], the NCI-CONNECT program is fostering progress by publishing guidelines [22] and hosting expert workshops [22, 23, 25, 27]. Therefore, our virtual MTB offered providers guidance and possibly offered patients reassurance that their case was being reviewed by a diverse group of specialists with the knowledge and tools needed to provide informed treatment suggestions, a critical benefit worth exploring in future studies [42].

This study has several limitations. The retrospective record review increased the potential for missing data. The early morning timing of the virtual MTB limited participation for some clinicians (e.g., those located on the US West). However, this limitation was addressed by hosting biweekly, alternating afternoon journal club sessions with optional time to accommodate presentation of cases by presenters in different time zones, including international. The virtual MTB relied heavily on a strong leader to ensure discussions remained on track [2]. Notably, our study lacked a dedicated follow-up assessment. Therefore, similar to the study by Khalafallah et al. (2021) [15], it remains uncertain if the suggestions were actually followed and, moreover, if they resulted in positive clinical outcomes. While Lutterbach et al. (2005) [43] identified that the recommendations made by their interdisciplinary brain tumor board colleagues had a high potential to be implemented, Pillay et al. (2016) [44], noted little evidence indicating that MTBs improve clinical outcomes. Therefore, future prospective, longitudinal work is needed to investigate the impact of virtual neuro-oncology MTBs on measures such as enrollment to clinical trials and patient satisfaction and outcome. Importantly, our group is currently developing a prospective, structured, and standardized data collection system, in collaboration with the NIH Center for Cancer Research Office of Information Technology, to address many of the limitations noted in this present study. Our group also plans to expand the educational component of the MTB by creating a repository of presented cases. Lastly, the virtual MTB did not include experts in bioinformatics or molecular biology, who could have contributed to the identification of actionable molecular alterations. However, in CNS tumors the presence of actionable genetic alterations may not be sufficient to predict a therapeutic response as there are other important factors such as the blood brain barrier penetration of the drugs being considered and the presence of intrinsic or acquired resistance.

## Conclusions

Our virtual multi-institutional MTB enabled expert review of challenging cases, regardless of geographic location of the patients. These novel remote meetings were particularly beneficial to formulate care plans for those with rare CNS tumors and AYAs, who face barriers to access specialized neuro-oncology providers. Our data also highlights the importance of advanced molecular testing in obtaining precise diagnoses and guiding treatment decisions, including discovering potentially actionable molecular targets with active clinical trial options. Ultimately, future work is needed to increase access to virtual MTBs and further refine administrative and logistical processes, with special attention to data protection and regulation of data access and sovereignty. Most importantly, virtual neuro-oncology MTBs open to external participants should remain an option post-pandemic to ensure equitable health care access.

### Supplementary Information

Below is the link to the electronic supplementary material.

Supplementary file1 (PDF 652 KB)

Supplementary file1 (DOCX 19.5 KB)

## Data Availability

The datasets generated during the current study are available from the corresponding author on reasonable request.
