# Supplementary material for: Virtual multi-institutional tumor board: a strategy for personalized diagnoses and management of rare CNS tumors
Source: J Neurooncol. 2024 Mar 1;167(2):349–59. doi: 10.1007/s11060-024-04613-6 (PMC11023967; doi:10.1007/s11060-024-04613-6)
Supplement: Supplementary file 1 — Supplementary file1 (PDF 652 KB) [file 11060_2024_4613_MOESM1_ESM.pdf]

Neuro-Oncology Virtual Tumor Board

NEURO-ONCOLOGY BRANCH

NIH NATIONAL CANCER INSTITUTE
Center for Cancer Research

The form must be submitted by end of business day on Monday the week you intend to present, at the latest. Tumor Board alternates each week between 8:00 am and 3:30 pm EST. Please contact Dr. Thomas Wall and/or Dr. Marta Penas-Prado to set the day of your presentation and receive further instructions.

TUMOR BOARD DETAILS

Day of Presentation (Month, Day, Year): ______________________

Presenter: ______________________ Hospital: ______________________

Specialty: ______________________

Date of Patient's Initial Primary CNS Tumor Diagnosis: ______________________

Diagnosis: ______________________

Reason for Discussion at Tumor Board:

DEIDENTIFIED Brief Narrative (Age, Gender, Handedness, Current KPS):
During the virtual tumor board, please be ready to present from your computer imaging studies, pathology slides and/or report - H&E, IHC, molecular. It is highly encouraged to present path slides, if available.

TUMOR BOARD DISCUSSION

Research Protocol(s) Available:

Suggestions:

The final plan will be decided by the treating physician who knows the patient well. Our suggestions are meant to contribute, but not dictate, the final management of cases presented at Tumor Board.
