# Supplementary material for: Virtual multi-institutional tumor board: a strategy for personalized diagnoses and management of rare CNS tumors
Source: J Neurooncol. 2024 Mar 1;167(2):349–59. doi: 10.1007/s11060-024-04613-6 (PMC11023967; doi:10.1007/s11060-024-04613-6)
Supplement: Supplementary file 2 — Supplementary file1 (DOCX 19.5 KB) [file 11060_2024_4613_MOESM2_ESM.docx]

**SUPPLEMENTARY TABLE 1**

| **Tumor Diagnosis Pre-Tumor Board** | **Newly Dx/Recurrent/Other** | **Molecular findings discussed at Tumor Board (4)** | **Methods molecular profiling** | **Did the diagnosis change based on molecular findings?** | **New Diagnosis or further sub-classification** | **Did molecular findings guide management suggestions?** | **Management suggestions from the Tumor Board (1)** |
| --- | --- | --- | --- | --- | --- | --- | --- |
| Infiltrating glioma tectum/pineal region | Newly Dx | **IHC:** IDH1 R132H neg, BRAFV600E neg, ATRX retained, H3K27me3 retained; **NGS:** no BRAF V600E mutation, Microsatellite status stable, Tumor mutation burden: 0 mut/Megabase | IHC, NGS panel (commercial) | No | N/A | Yes | Observation alone |
| Meningioma (WHO grade 1 vs grade 2) with extra-CNS dissemination | Recurrent | **NGS:** NF2 mutation, Microsatellite status stable, Tumor mutation burden: 0.5 mut/Megabase; **RNAseq:** SMARCB1 underexpressed, CCND1 and EGFR overexpressed; **WES:** germline pathogenic mutation SDHA gene (c.91 C>T); **DNA methylation:** chromosome 1p loss in copy number plot, methylome suggestive of high recurrence risk (RFS score 0.32-0.21) | NGS panel (commercial); RNA seq (commercial); WES (commercial); DNA methylation (NIH) | Yes (2) | Sub - classification - DNA Methylation shows Higher Risk | Yes | Genetic counseling |
| Spinal MYCN-amplified anaplastic ependymoma | Newly Dx | MYCN amplification | NGS panel (not specified) | No | N/A | Yes | Best approach for radiation planning; offered case presentation at recurrence to discuss standard or experimental options |
| Pituitary region high-grade neuroendocrine tumor/Primitive neuroepithelial tumor (PNET) | Newly Dx | **NGS:** SMARCB1 mutation; **DNA methylation:** ATRT, Myc subtype | NGS panel (NIH); DNA methylation (NIH) | Yes | New Diagnosis with Sub - classification (ATRT myc subtype) | Yes | Craniospinal Radiation followed by multiagent cytotoxic chemotherapy such as ICE |
| Spinal Diffuse Midline Glioma, H3K27M mutant | Newly Dx | H3K27M mutation | NGS panel (not specified) | No | N/A | Yes | Radiation and chemotherapy options discussed. Suggested radiation field: postoperative bed plus generous margin, with careful planning to allow for additional radiation to other CNS segments in the future. Consider proton radiation therapy to decrease toxicities. Use of concurrent and adjuvant chemotherapy (temozolomide) can be considered given the typically aggressive course of these tumors |
| Brainstem infiltrating glioma | Newly Dx | **NGS:** No IDH1, IDH2 or H3K27M mutations; BRAF fusion; **DNA methylation:** pilocytic astrocytoma | NGS panel (not specified); DNA methylation (NIH) | Yes | New Diagnosis (Pilocytic astrocytoma) | Yes | Observation alone |
| Medulloblastoma | Recurrent | **NGS**: IDH1 mutation, PIK3CA mutation, KDM6A mutation, TERT mutation, Microsatellite status stable, Tumor mutation burden 3.1 mut/Megabase; **DNA methylation:** medulloblastoma, SHH subgroup | NGS panel (NIH); DNA methylation (NIH) | Yes | Sub-classification (SHH subtype) | Yes | Possible targeted drugs (SMO inhibitors); re-irradiation |
| High-grade astrocytoma with molecular features of glioblastoma | Recurrent | No IDH1 or IDH2 mutations, TERT mutation, NF1 mutation, Tumor mutational burden 7 mut/Megabase | NGS panel (not specified) | No | N/A | Yes | Explore potential trials at other sites; second-line cytotoxic agents; checkpoint inhibitor; potential future trial at NIH |
| High-grade neuro-epithelial tumor with MN1 alteration (HGNET-MN1) | Recurrent | **NGS:** no clinically relevant variants, MN1::BEND2 fusion; **DNA methylation:** CNS high-grade neuroepithelial tumor with MN1 alteration | NGS panel (NIH); DNA methylation (NIH) | Yes | New Diagnosis (HGNET-MN1) | Yes | Obtain further molecular testing on the most recent tumor sample; discussed treatments options including re-irradiation, multiagent cytotoxic chemotherapy, temozolomide or targeted drugs if an actionable mutation is found |
| Intraventricular high-grade glioma with ependymal and sarcomatous differentiation | Newly Dx | No IDH1, IDH2, BRAF, or TERT mutations; NF2 loss, CDKN2A mutation, CCNE1 amplification; Unmethylated MGMT | NGS panel (not specified); MGMT methylation | No | N/A | Yes | Obtain DNA methylation and L1CAM IHC testing; recommended against use of concurrent temozolomide during radiation due to atypical features for GBM and unmethylated MGMT |
| Glioblastoma, IDH mutant | Other (Recurrent vs Tx Effect) | IDH mutation | NGS panel (not specified) | No | N/A | Yes | Resection not recommended; future candidate for a trial for IDH mutated tumors at the NIH if progressive disease |
| Rosette-forming glioneuronal tumor, WHO Grade I | Other (rule out recurrence) | **NGS:** FGFR1 mutation; **DNA methylation:** RFGNT | NGS panel (NIH); DNA methylation (NIH) | No | N/A | Yes | Imaging changes felt to be due natural history of RFGNT and not to tumor growth; recommended observation alone |
| Multifocal anaplastic astrocytoma, WHO Grade III | Other (rule out recurrence) | **NGS:** IDH1 mutation; **DNA methylation:** IDH mutant glioma | NGS panel (NIH); DNA methylation (NIH) | Yes | Change in grade, subclassification (IDH mutant) | No | Recommended full spine MRI, potential resection, and radiation |
| Ependymoma, Posterior Fossa-B with spinal dissemination | Recurrent | **NGS:** no clinically relevant variants; **DNA methylation:** ependymoma, posterior fossa B | NGS panel (NIH); DNA methylation (NIH) | Yes | Subclassification (PF-B) | No | Obtain short-term follow-up imaging; given slow growth with no new symptoms and minimal mass effect on the neural elements, no intervention was recommended |
| Spinal MYCN-amplified anaplastic ependymoma disseminated to the brain | Recurrent | **NGS:** MYCN amplification; **DNA methylation:** noncontributory | NGS panel (NIH); DNA methylation (NIH) | Yes | Sub-classification (MYCN) | No | Surgical resection of brain lesions recommended |
| Posterior fossa anaplastic ependymoma, WHO Grade III with spinal dissemination | Recurrent | **NGS:** no clinically relevant variants; **DNA methylation:** ependymoma, posterior fossa B | NGS panel (NIH); DNA methylation (NIH) | Yes | Sub-classification (PF-B) | No | Consider resection and off-label PD1 inhibitor |
| Intraventricular myxoid mesenchymal tumor with EWSR1-ATF1 fusion and LMD | Newly Dx | **NGS:** EWSR1-ATF1 fusion; **DNA methylation**: no match in brain or sarcoma classifier | NGS panel (NIH); DNA methylation (NIH) | No | N/A | Yes | Suggested whole brain radiation with careful planning to allow for additional radiation to the spine in the future, if needed. Recommended against chemotherapy due to poor penetration of sarcoma regimens into the brain |
| Spinal pilocytic astrocytoma with BRAF fusion | Newly Dx | **NGS:** BRAF fusion; **DNA methylation:** pilocytic astrocytoma | NGS panel (NIH); DNA methylation (NIH) | No | N/A | Yes | Suggested participation in trial using a RAF inhibitor or off-label use of MEK inhibitor |
| Spinal malignant melanocytic schwannian tumor | Recurrent | **NGS:** PRKAR1A and TP53 mutations, Microsatellite status stable, Tumor Mutation burden 1.57 mut/Megabase; **DNA methylation:** schwannoma | NGS panel (commercial and NIH); DNA methylation (NIH) | No | N/A | Yes | Suggested germline testing for Carney complex |

FOOTNOTES:

1) Level of evidence for all tumor board suggestions was based on Non-randomized studies, Case series, and/or Expert Opinion.

2) RFS score reference - Nassiri, F., et al., DNA methylation profiling to predict recurrence risk in meningioma: Development and validation of a nomogram to optimize clinical management. Neuro-oncology, 2019. 21(7): p. 901-910

3) **Dx** (Diagnosis), **ICE** (ifosfamide/carboplatin/etoposide), **IHC** (Immunohistochemistry), **LMD** (leptomeningeal dissemination), **NGS** (Next Generation Sequencing), **Tx** (Treatment), **RFGNT** (Rosette-forming glioneuronal tumor), **WES** (Whole Exome Sequencing).

4) All molecular alterations listed in NGS were variants of clinical or pathological significance.
